# MiRNA expression profiles in healthy OSAHS and OSAHS with arterial hypertension: potential diagnostic and early warning markers

**DOI:** 10.1186/s12931-018-0894-9

**Published:** 2018-10-02

**Authors:** Xiuping Yang, Xun Niu, Ying Xiao, Kun Lin, Xiong Chen

**Affiliations:** Department of Otolaryngology, Union Hospital, Tongji Medical College, Huazhong University of Science and Technology, Wuhan, 430022 China

**Keywords:** OSAHS, miRNAs, Complications, Marks, Diagnostic

## Abstract

**Background:**

Obstructive sleep apnea-hypopnea syndrome (OSAHS) is prone to being complicated with various cardiovascular, cerebrovascular and metabolic conditions. OSAHS, due to its multifactorial nature, entails individualized and comprehensive treatment. So far, no well-established diagnostic criteria for the disease are available. In recent years, miRNA has been shown to be a sensitive biomarker suggestive of the progression and prognosis of many diseases. In this study, we examined some serum miRNAs in healthy OSAHS (OSAHS patients without complication) and OSAHS with arterial hypertension, with an attempt to understand the potential effects on the disease, improve the diagnosis of OSAHS and find OSAHS-related diagnostic markers.

**Methods:**

Against various diagnostic criteria, participants were divided into three groups: healthy OSAHS, OSAHS with arterial hypertension and healthy controls. Their serum miRNA profiles were assessed by microarray technology, and then differentially expressed miRNAs were verified by quantitative real-time PCR (qRT-PCR). The receiver operating characteristic (ROC) curves of miRNAs were constructed and the areas under the curve (AUC) were calculated. Meanwhile, the miRNAs were subjected to logistic regression analysis. The target genes were bioinformatically assessed, their functions and signaling pathways further determined and eventually an miRNA-gene network was established.

**Results:**

Analysis with the miRNA array exhibited that, compared with the control group, 12 differentially expressed miRNAs were found in healthy OSAHS, and 33 were found in OSAHS with arterial hypertension. The expression of miR-126-3p, let-7d-5p, miR-7641 and miR-1233-5p, miR-320b, miR-145-5p, miR-107, miR-26a-5p were validated by using qRT-PCR. Bioinformatics analysis predicted that the potential target genes of these miRNAs might be involved in metabolism, and the regulation of endothelial cells and nervous system. Moreover, the ROC analysis showed that the using miR-145-5p and let-7d-5p in combination can identify the healthy OSAHS, presence of miR-126-3p, miR-26a-5p and miR-107 was well indicative of OSAHS with arterial hypertension.

**Conclusions:**

A cluster of dysregulation miRNAs have been found to be involved in the development of OSAHS patients. Moreover, these miRNAs might be used to be potential diagnostic and early warning markers.

## Background

OSAHS is a complex, multifactorial disorder known to affect, with varying degrees, millions of people worldwide [1]. The clinical manifestations and therapeutic effects vary considerably with the stage of the disease. Therefore, early diagnosis and treatment are crucial to treatment efficacy and the prevention of complications. The apnea hypopnea index (AHI), the sum of apneas and hypopneas per hour of sleep, has been extensively used as a criterion for the diagnosis and assessment of OSAHS [2]. Nonetheless, the incidence of OSAHS-related complications tend to vary substantially even in patients with similar AHI. Obviously, efficacy of the treatment vary with patients even with similar AHI. In fact, AHI, as a single parameter, is not a reliable measure of the severity of OSAHS [3–5]. Ideally, any treatment decision about OSAHS should be based on stratification of OSAHS symptoms. This “ideal OSAHS decision” should not be made against AHI alone. MiRNAs serve as important regulators in multicellular organisms and can regulate protein expression by blocking mRNA translation [6]. By coordinating the expression of multiple genes, miRNAs establish a broad network that regulates various biological processes, such as cellular differentiation, proliferation, apoptosis and metabolism. In humans, miRNAs are ubiquitous in tissues or body fluids and are stably expressed in blood [7, 8]. Changes in miRNAs may precede the appearance of physical symptoms of certain diseases [9, 10]. The altered expression of miRNAs have been found in a wide array of pathologies, including cancers, diabetes, hypoxia and hypertension [11–14]. Many studies have demonstrated that miRNAs played pivotal parts in the maintenance of metabolic homeostasis and stable blood pressure [15, 16]. Therefore, in-depth study on their roles can help us better understand the mechanisms of diseases and provide new targets for their treatment.

As we know, OSAHS involves multiple organs and systems, such as cardiovascular systems. About one-third of hypertensive patients have OSAHS, and about half of OSAHS patients have hypertension [17]. A multitude of studies have demonstrated the association between OSAHS and hypertension, especially the resistant hypertension [18, 19]. Individuals suffering from both OSAHS and hypertension tend to have increased cardiovascular risk.

In this study, we examined some diagnostic evidence and identified a set of warning signs for the progression of the disease and compared healthy OSAHS and OSAHS with arterial hypertension, with an attempt to provide diagnostic evidence and early warning indicators for a better diagnosis of OSAHS.

## Methods

### Ethics statement

This study was approved by the Ethics Committee of the Union Hospital of Tongji Medical College, Huazhong University of Science and Technology, Wuhan, China, and written informed consent was obtained from all participants.

### Study participants

The subjects were those who visited the Department of Otorhinolaryngology, the Union Hospital of Tongji Medical College, Huazhong University of Science and Technology, Wuhan, China, due to snoring or apnea from March 2017 to November 2017. Healthy controls were recruited from the Health Screening Center of the same hospital and didn’t have any medical diseases or snoring.

All participants received overnight polysomnography (PSG), OSAHS patients and normal subjects were confirmed on the basis of American Academy of Sleep Medicine (AASM) Guidelines. Meanwhile, all participants underwent physical checkups, blood pressure measurement and tests of blood biochemistry.

The criteria for exclusion included: (1) presence of other sleep disorders (International Classification of Sleep Disorders (ICSD-II) diagnosis) and history of having received treatment for sleep related breathing disorders; (2) other diseases such as central nervous system diseases, cardiopathy, diabetes, renal disease, thyroid disease, cancer, ongoing infections; (3) old age (> 60 year-old), young age (< 24 year-old), or being pregnant.

They were divided into 3 groups, each group having 20 subjects: (1) healthy controls (AHI < 5, without hypertension, hyperglycemia or dyslipidemia), (2) healthy OSAHS (AHI > 5, without hypertension, hyperglycemia or dyslipidemia) and (3) OSAHS with arterial hypertension (AHI > 5, with hypertension without hyperglycemia or dyslipidemia). In each group, 5 subjects were subjected to microarray and the other 15 subjects received qRT-PCR for validation.

### Blood sample collection

Blood samples were collected from all the participants at AM 8:00 after receiving full-night in-laboratory polysomnography and an overnight fasting. For miRNA studies, blood was harvested into an inert separation gel vacuum procoagulant tube. Within 60 min of blood collection, the blood was centrifuged at 3000 g for 10 mins to obtain serum. The supernatant was transferred to RNase-free Eppendorf tubes and then stored at − 80 °C until RNA extraction.

### MiRNA isolation and expression analysis by microarray

Total RNA, including miRNA, was extracted from 15 frozen serum by using QIAGEN RNeasy Mini Kit (217,004; QIAGEN, Germany) according to the manufacturer’s instructions. RNA concentration was determined on an ND-2000 spectrophotometer (Nanodrop™, Thermo Fisher Scientific, USA) at 260 nm, 280 nm and RNA integrity was determined by RNA gel electrophoresis. Small isolated RNAs was processed with the FlashTag®Biotin HSR RNA labeling kit (Affymetrix, USA) and was subsequently subjected to hybridization in the GeneChip® Hybridization Oven 645 (Affymetrix, USA). Washing and scanning were respectively conducted on the GeneChip® Fluidics Station 450 (Affymetrix, USA) and the GeneChip® Scanner 3000 7G (Affymetrix, USA) by following instructions. The data were analyzed against Affymetrix Expression Console-1_4_0 (EC 5.0) by using Robust Multichip Average (RMA) as normalization method.

### qRT-PCR verification of miRNA array results

Of the significantly differentially expressed miRNAs (there was at least a two-fold level change between the two groups and *p* value < 0.05) as found by the microarray, the miRNAs found in both healthy OSAHS and in OSAHS with arterial hypertension and in those that have been reportedly associated with mental disorder, endothelial dysfunction, oxidative stress or angiogenesis, were selected. Those miRNAs were verified in 45 blood samples by qRT-PCR. Total RNA was extracted according to the instructions of miRNeasy serum kit (217,184; QIAGEN, Germany). Moreover, due to the unavailability of stable internal control in the serum, *C. elegans* miR-39 miRNA mimic, the miRNeasy serum/plasma spike-in control (219,610; QIAGEN, Germany), was used to monitor miRNA purification and amplification. cDNA was synthesized from total RNA using the miScript II RT kit (218,161; QIAGEN, Germany) and qRT-PCR was performed in a Lightcycle 480 RT-PCR system (Roche Diagnostics Ltd., Rotkreuz, Switzerland) using the miScript SYBR Green PCR kit (218,073; QIAGEN, Germany). The thermal cycle profile was as follows: an initial activation of 15 min at 95 °C, and 45 cycles of denaturation (15 s at 94 °C), annealing (30 s at 55 °C) and extension (30 s at 70 °C). The specificity and identity of PCR products were evaluated by melting curve analysis, with cel-miR-39 used as a normalization standard for miRNAs. The relative levels of each miRNAs were determined by 2^−[Ct(miRNA)-Ct(cel-miR-39)]^. Primer sequences are listed in Table 1.

**Table 1:** Primers for qRT-PCR

miRNA	Forward primer squence
miR-126-3p	TGCGCTCGTACCGTGAGTAATA
let-7d-5p	TGCGCAGAGGTAGTAGGTTGCA
miR-7641	TGCGCTTGATCTCGGAAGC
miR-1233-5p	TGCGCAGTGGGAGGCCAGGGCA
miR-320b	TGCGCAAAAGCTGGGTTGAGAG
miR-145-5p	TGCGCGTCCAGTTTTCCCAGGAA
miR-107	TGCGCAGCAGCATTGTACAGGGC
miR-26a-5p	TGCGCTTCAAGTAATCCAGGAT
cel-miR-39	TGCGCTCACCGGGTGTAAATCA

The reverse primer was the miScript Universal Primer of the miScript SYBR Green PCR Kit (218,073; QIAGEN, Germany)

### Bioinformatic analysis of proven miRNAs

We identified the biological processes that might be affected by these differentially expressed miRNAs by employing genomic enrichment analysis of regulated target genes. On the basis of the two groups of miRNAs obtained from the analysis, we used the miRanda and TargetScan databases to predict the target genes of the differentially expressed miRNAs. Next, the function of the differentially expressed genes were analyzed by using GO analysis [20]. Meanwhile, KEGG pathways analysis was applied to predict the significant signaling pathways of the target genes, according to the microarray data [21, 22]. Then, we obtained the intersection target genes identified by GO and KEGG prediction. Besides, we constructed an miRNA-gene-network according to the targeting relationship between the miRNAs and these genes.

### Statistical analysis

Normally distributed data were presented as means ± SEM, non-normally distributed data were expressed as medians (the first and third quartile values), and qualitative variables were given as frequencies and percentages. Possible differences in demographic and clinical variables among the groups were evaluated by one-way analysis of variance (ANOVA) and Kruskal-Wallis. For microarray analysis, we adopted the Random variance model modified *t*-test to filter the differentially expressed miRNAs between patients and healthy controls. We distinguished the different miRNAs by using the significance analysis and FDR analysis. Fisher’s exact test and χ2 test were employed to classify the GO categories and signaling pathways, and the false discovery rate (FDR) was calculated to calibrate the *p* value. For the results of qRT-PCR, independent sample Student *t*-test and Mann-Whitney U tests were performed for statistical analysis. To further determine the prediction values of the selected miRNAs, ROC curves were constructed among the three groups. The AUC was calculated, and the optimum sensitivity and specificity were determined by Youden index. Logistic regression models were generated to assess the value of the differentially expressed miRNAs in combination in the identification of OSAHS patients. Variables were included in the model if they exhibited statistically significant contributions as shown by the likelihood ratio test. The AUC was used to determine the maximum differentiating ability of the model. Variables which had no statistically significant contributions to the model but could increase its AUC were also selected. All analyses were performed using GraphPad Prism (version 5.0; GraphPad Software) and statistical software package SPSS (version 22.0, SPSS Inc., Chicago, IL, USA). All tests were two-sided and a *p* value < 0.05 was considered to be statistically significant.

## Results

### Demographic data and clinical characteristics of participants

All participants enrolled were Chinese. Table 2 details the clinical characteristics, covering body mass index (BMI), PSG, and biochemical data. The age, BMI, gender, smoking and drinking were all matched among the three subgroups.

**Table 2:** The clinical characteristics of participants

	Training set				Validation set			
	Healthy controls	Healthy OSAHS	OSAHS with arterial hypertension	*P* value	Healthy controls	Healthy OSAHS	OSAHS with arterial hypertension	*P* value
Number	5	5	5		15	15	15	
Age, (years)	39.4 ± 1.69	40.6 ± 3.17	43.8 ± 1.24	0.37	43.4 ± 2.1	43 ± 2.51	43.6 ± 2.33	0.98
Male, n (%)	5 (100%)	5 (100%)	5 (100%)	–	11 (73.33%)	13 (86.67%)	12 (80%)	0.68
BMI, (kg/m^2^)	23.5 ± 0.42	25.18 ± 1.18	25.99 ± 0.38	0.1	24.57 ± 0.46	25.56 ± 0.38	26.16 ± 0.56	0.07
Smoker, n (%)	2 (40%)	1 (20%)	1 (20%)	0.76	1 (6%)	2 (13%)	4 (27%)	0.32
Drinker, n (%)	0	2 (40%)	2 (40%)	0.3	2 (13%)	4 (27%)	7 (47%)	0.13
Cholesterol, (mmol/L)	4.42 ± 0.27	4.4 ± 0.14	3.86 ± 0.4	0.35	4.37 ± 0.19	4.42 ± 0.16	4.4 ± 0.24	0.99
Triglycerides, (mmol/L)	0.87 ± 0.05	1.44 ± 0.15	1.56 ± 0.25	0.04	1.23 ± 0.13	1.5 ± 0.13	1.48 ± 0.12	0.25
HDL, (mmol/L)	1.36 ± 0.14	0.95 ± 0.09	0.86 ± 0.09	0.02	1.26 ± 0.06	1 ± 0.03	1.01 ± 0.05	< 0.001
LDL, (mmol/L)	2.55 ± 0.15	2.88 ± 0.14	2.32 ± 0.35	0.28	2.45 ± 0.17	2.8 ± 0.13	2.85 ± 0.2	0.2
Glucose, (mmol/L)	4.4 ± 0.1	4.54 ± 0.15	4.72 ± 0.14	0.25	4.78 ± 0.12	4.93 ± 0.12	4.92 (4.7,5.2)	0.35
HbA1c, (%)	5.34 ± 0.17	5.56 ± 0.13	5.52 ± 0.08	0.47	5.33 ± 0.11	5.55 ± 0.06	5.46 ± 0.09	0.21
ESS	3 ± 1.48	13.8 ± 2.71	13.6 ± 2.09	< 0.001	7.27 ± 1.43	13.13 ± 1.65	8.6 ± 1.21	< 0.05
AHI, (events/hour)	1.96 ± 0.52	39.27 ± 6.14	45.27 ± 8.2	< 0.001	4.52 ± 0.56	47.44 ± 4.92	43.46 ± 5.25	< 0.001
Mean SaO_2_, %	97 (96,97)	92.8 ± 1.59	93.8 ± 0.66	< 0.05	96 (95.97)	92.47 ± 0.97	94 ± 0.5	< 0.001
Minimum SaO_2_, %	92.6 ± 1.08	69.4 ± 3.04	74.6 ± 4.61	< 0.001	90.4 ± 0.58	74 (60,82)	72.87 ± 2.02	< 0.001

*OSAHS* Obstructive sleep apnea hypopnea syndrome, *BMI* Body mass index, *AHI* Apnea hypopnea index, *SaO*_*2*_ Oxygen saturation, *ESS* Epworth Sleepiness Scale, *HDL* High density lipoprotein, *LDL* Low density lipoprotein, *HbA1c* Glycosylated hemoglobin

### Differential expression of miRNAs

**Fig. 1:**
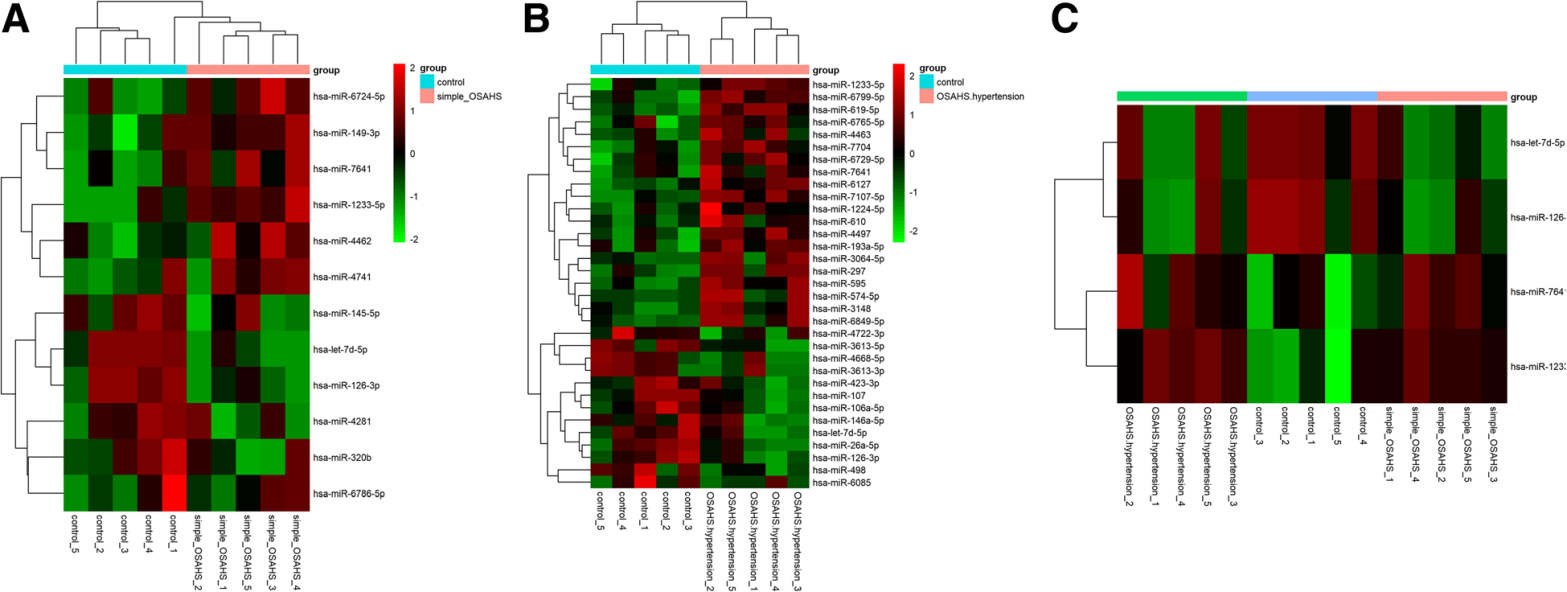
Hierarchical clustering plot (heatmap) of differentially expressed miRNAs in healthy controls (*n* = 5), healthy OSAHS (n = 5), OSAHS with arterial hypertension (n = 5). **a** Healthy OSAHS had dysregulated miRNAs compared to healthy controls (*P* < 0.05); (**b**) OSAHS with arterial hypertension had dysregulated miRNAs compared to healthy controls (*P* < 0.05); (**c**) 2 overlap miRNAs were up-regulated and the other 2 overlap miRNAs were down-regulated. The right bar represents the signal intensity of miRNA expression from − 2 (green) to 2 (red). Green: down-regulation; red: up-regulation; black: no change

The microarray revealed that miRNAs experienced changes in healthy OSAHS and their counterparts with arterial hypertension as compared to controls. Compared to the healthy controls, 12 miRNAs were deregulated (*P* < 0.05) in healthy OSAHS. Among them, 6 were up-regulated and 6 were down-regulated (*P* < 0.05) (Fig. 1a). In OSAHS with arterial hypertension, 33 miRNAs were deregulated (*P* < 0.05), compared with the healthy control group. Of them, 20 were up-regulated and 13 down-regulated (*P* < 0.05) (Fig. 1b). Of note, 4 miRNAs were similarly deregulated in healthy OSAHS and OSAHS with arterial hypertension (Fig. 1c). The results were submitted to NCBI’s Gene Expression Omnibus (GEO).

### qRT-PCR identification of differentially regulated miRNAs

**Fig. 2:**
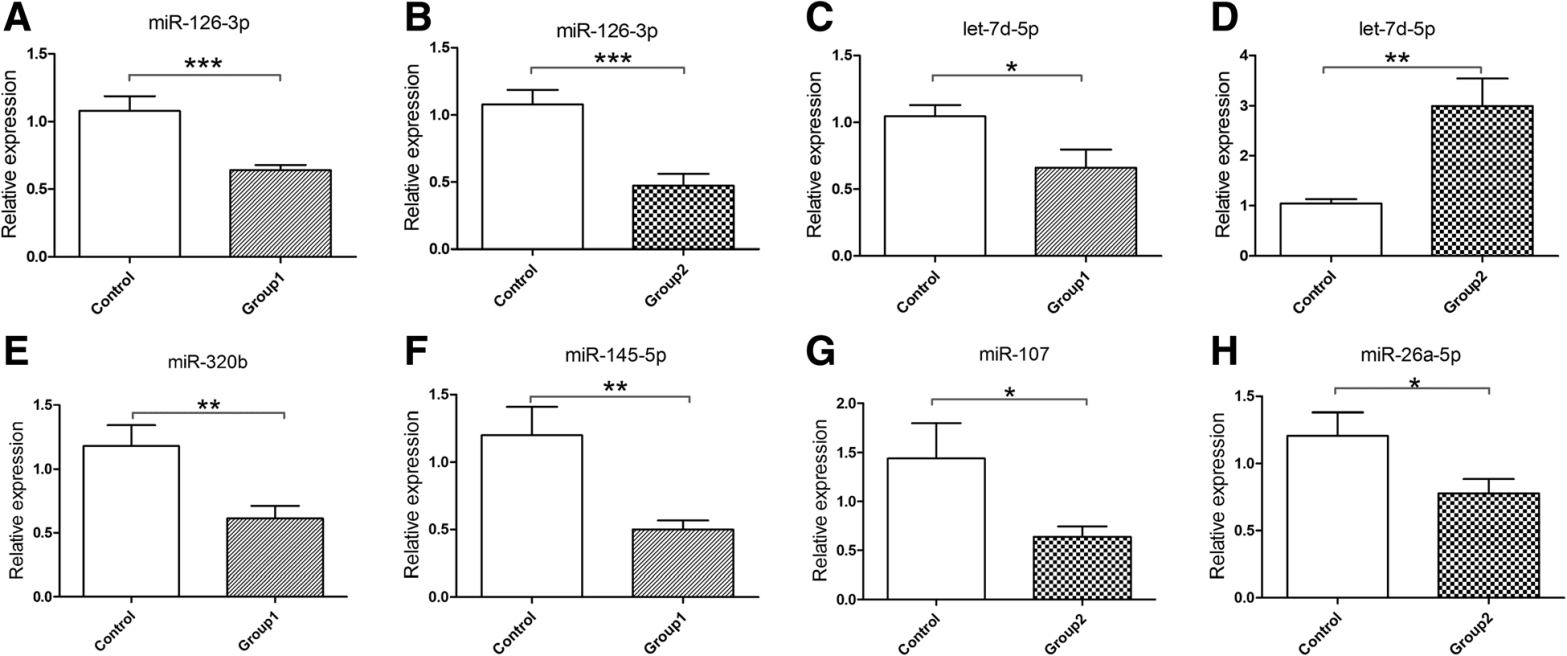
qRT-PCR validation of serum miRNA levels in three groups (Group 1: healthy OSAHS, Group 2: OSAHS with arterial hypertension). The relative expression level of (**a**, **b**) miR-126-3p, (**c**, **d**) let-7d-5p, (**e**) miR-320b, (**f**) miR-145-5p, (**g**) miR-107, (**h**) miR-26a-5p. The data are expressed as mean ± SEM. *P < 0.05, **P < 0.01, ***P < 0.001, NS: no significant changes

As aforementioned, we chose 6 miRNAs for qRT-PCR validation. Of them, 4 were overlapping miRNAs from healthy OSAHS and OSAHS with arterial hypertension (miR-126-3p, let-7d-5p, miR-7641 and miR-1233-5p), two were from OSAHS with arterial hypertension (miR-107, miR-26a-5p). Despite their low fold-change of 1.45 and 1.2, miR-145-5p and miR-320b were also chosen from healthy OSAHS, because, according to the selection criteria, only overlapping miRNAs would be selected in healthy OSAHS. Meanwhile, these two miRNAs had been considered to be potential biomarkers indicative of cardiovascular conditions and hypoxia. Thus, 8 miRNAs were selected for validation by qRT-PCR. Only miR-126-3p was verified to be down-regulated in both OSAHS groups (Fig. 2a, b) (*P* < 0.05). Moreover, let-7d-5p was down-regulated only in healthy OSAHS (Fig. 2c, d) (*P* < 0.05). Significant down-regulation of other 4 miRNAs (miR-320b, miR-145-5p, miR-107, miR-26a-5p) was also confirmed (Fig. 2e, f, g, h) (*P* < 0.05). The expression levels of miR-7641 and miR-1233-5p were too low to be appreciable in the three groups (data not show).

### Microarray-based bioinformatical prediction

**Fig. 3:**
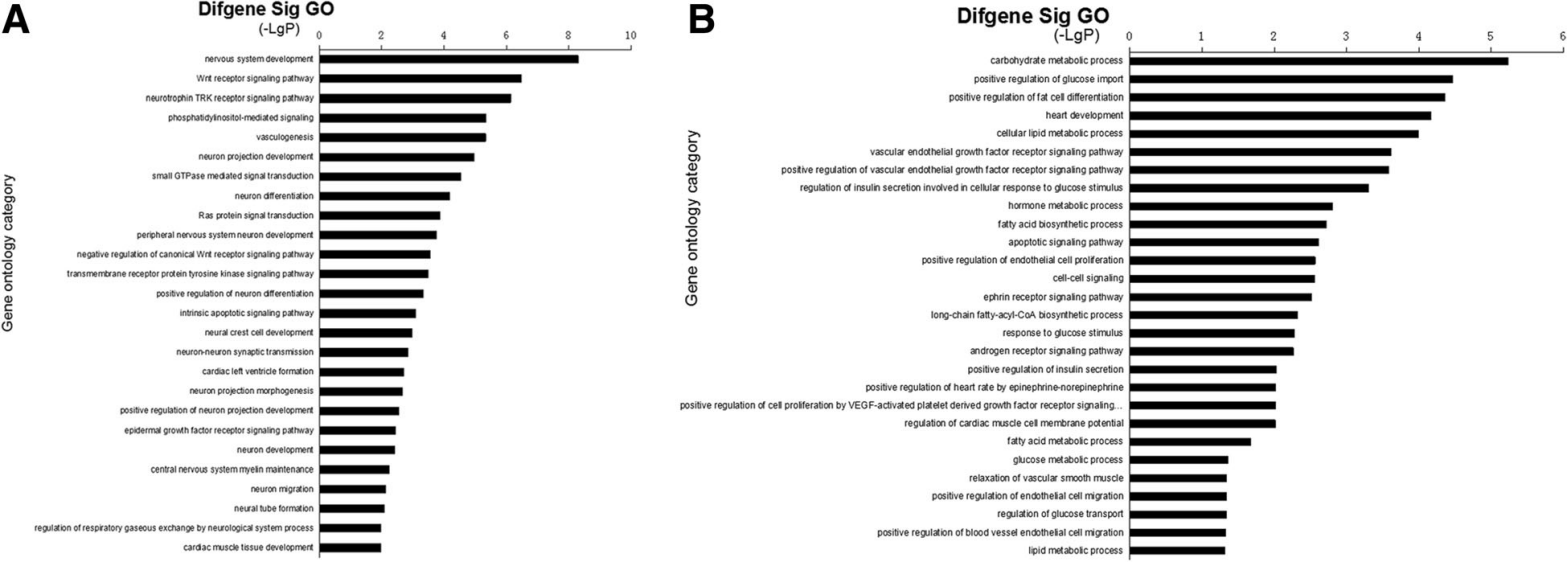
GO analysis for target genes of validated miRNAs. **a** the significant GOs of the genes targeted by the validated miRNA in healthy OSAHS. **b** the significant GOs of the genes targeted by the validated miRNA in OSAHS with arterial hypertension. -LgP is the negative logarithm of the *P* value. The larger the -LgP value, the smaller the *P* value, and the more important the roles of GOs

**Fig. 4:**
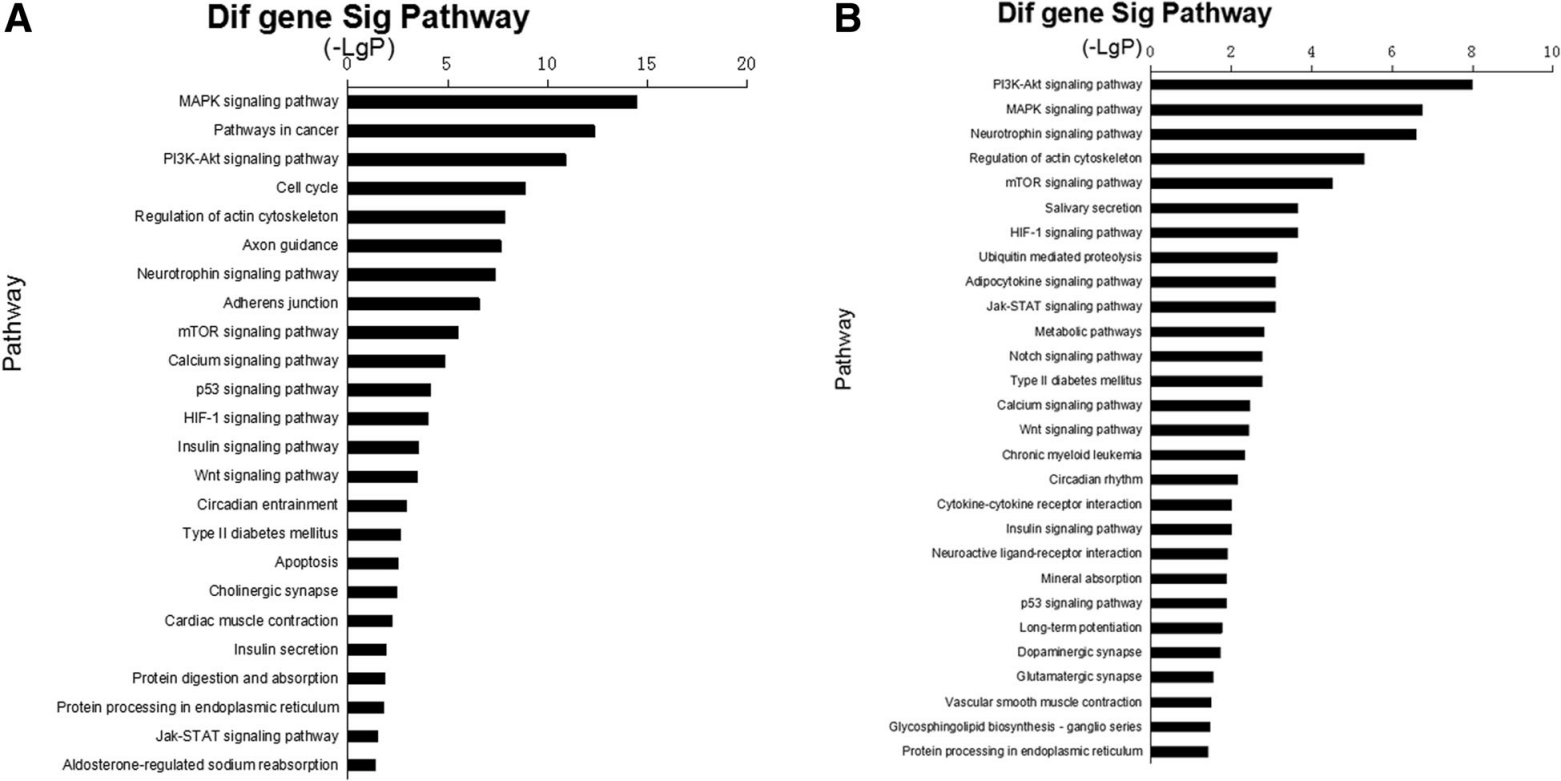
Pathway analysis for target genes of validated miRNAs. **a** the significant pathways of the genes targeted by the validated miRNA in healthy OSAHS. **b** the significant pathways of the genes targeted by the validated miRNA in OSAHS with arterial hypertension. -LgP is the negative logarithm of the *P* value. The larger the -Lg*P* value, the smaller the *P* value, and the more important the roles of pathways

**Fig. 5:**
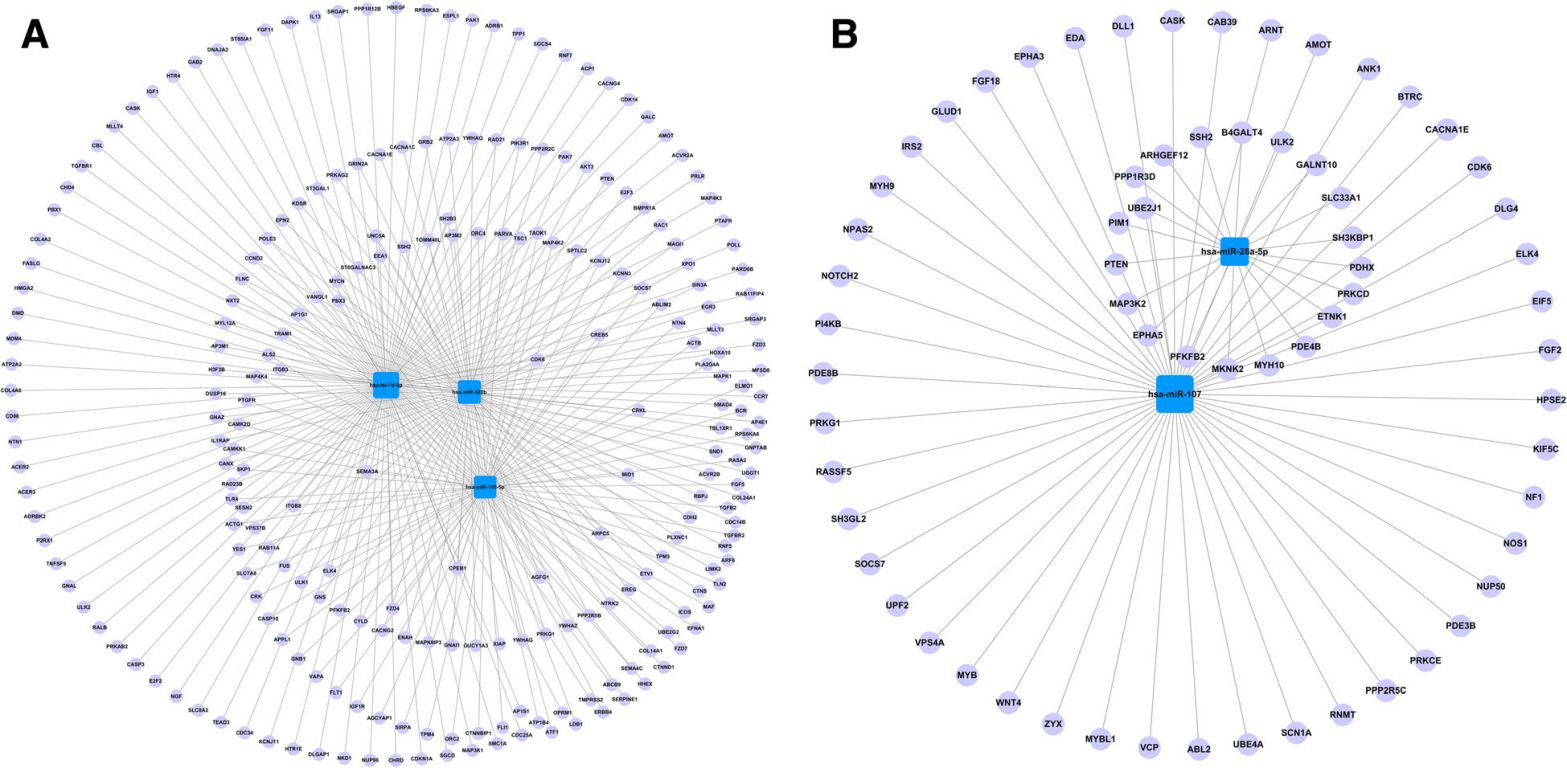
miRNA-gene-network. The mauve circles represent genes, the blue squares represent miRNAs, and gray lines represent the relationship between miRNA and genes. The size of the blue square is dependent on the number of genes regulated by an miRNA. **a** miRNAs-gene-network for the healthy OSAHS; (**b**) miRNAs-gene-network for OSAHS with arterial hypertension

From the miRanda and TargetScan databases, we retrieved 959 mRNAs corresponding to miRNAs we verified in healthy OSAHS and 537 mRNAs corresponding to miRNAs we verified in OSAHS with arterial hypertension. Next, these target genes were subjected to GO analysis and KEGG pathway analysis. In the healthy OSAHS group, GO analysis revealed these target genes involved 26 GOs, which were related to nervous systems, cardiovascular system, cancer and signal transduction (*P* < 0.01) (Fig. 3a). At the same time, 28 GOs were demonstrated to be regulated in the OSAHS with arterial hypertension (*P* < 0.01) (Fig. 3b). These GOs were implicated in metabolism, hormone secretion, vascular endothelial growth, signal transduction. Similarly, the pathway analysis showed that 25 pathways were regulated in the healthy OSAHS and 28 pathways in the OSAHS with arterial hypertension (*P* < 0.05) (Fig. 4a, b). Furthermore, the miRNA-gene-network consisting of the core miRNAs and key target genes revealed the relationship between miRNA and target genes (Fig. 5a, b).

### Diagnostic accuracy of the selected miRNAs

**Fig. 6:**
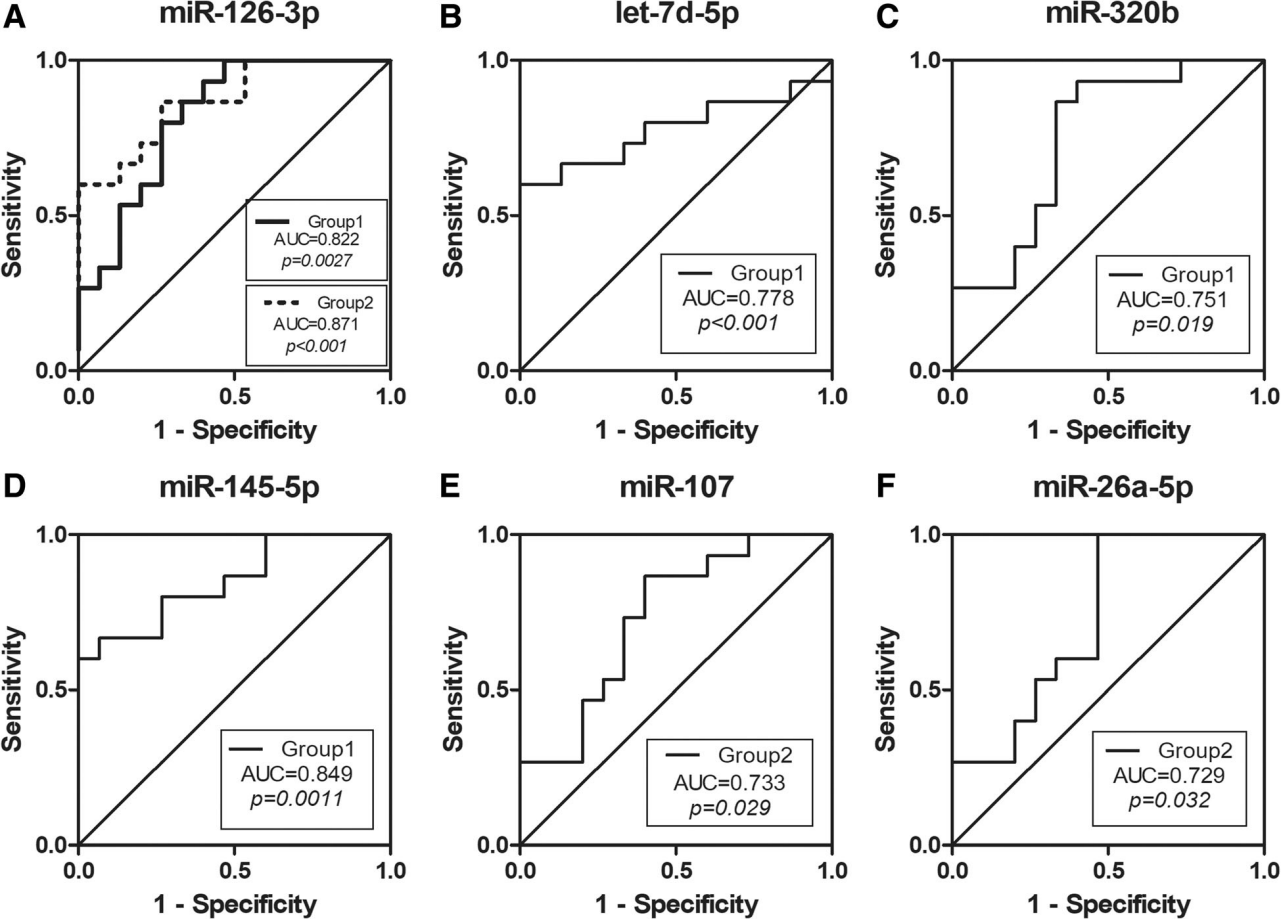
Receiver operating characteristic (ROC) curves analysis of serum miRNAs for three groups (Group 1: healthy OSAHS, Group 2: OSAHS with arterial hypertension). **a** miR-126-3p, (**b**) let-7d-5p, (**c**) miR-320b, (**d**) miR-145-5p, (**e**) miR-107, (**f**) miR-26a-5p. The area under the curve (AUC) was also shown

**Fig. 7:**
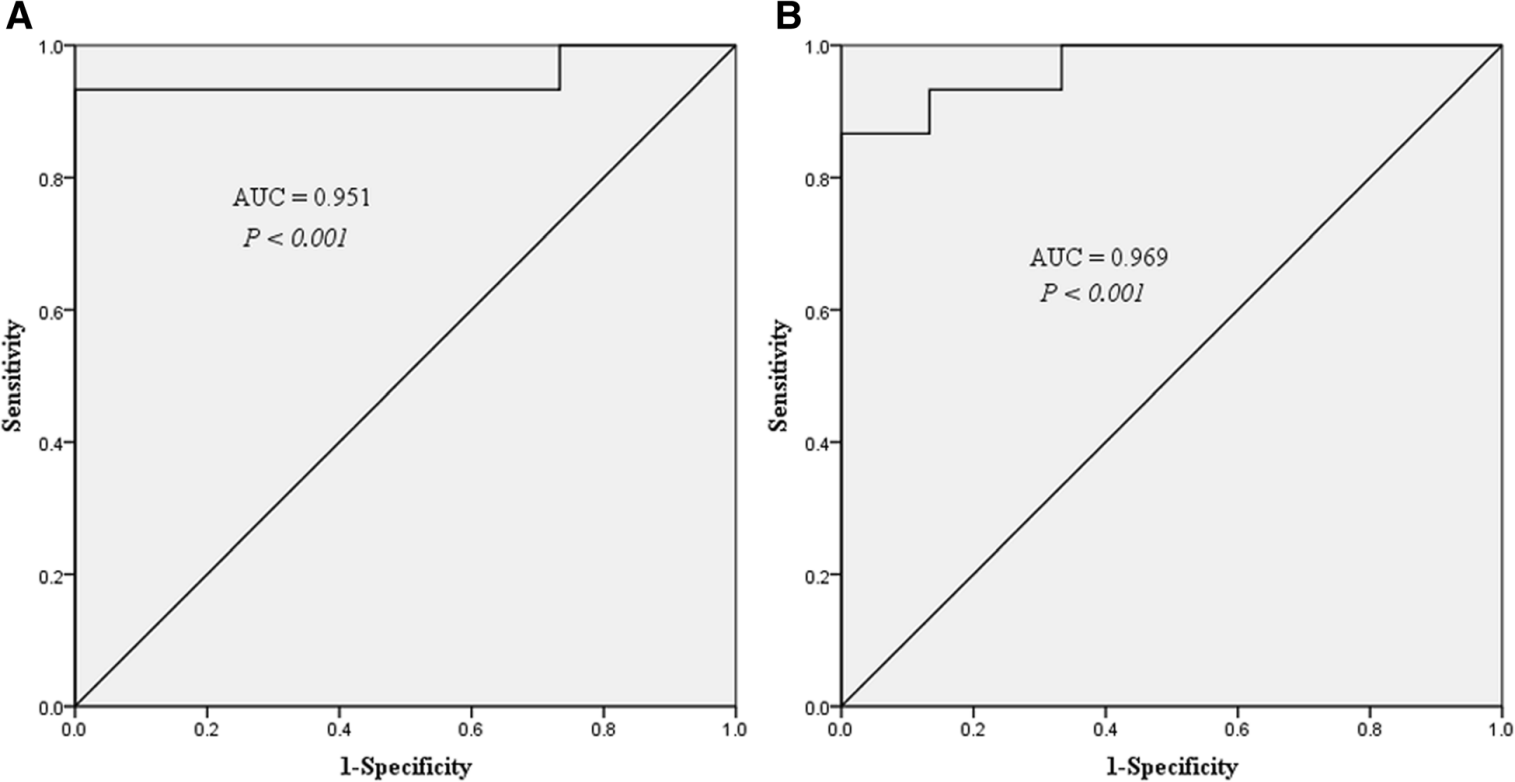
Receiver operating characteristic (ROC) curves for multivariate logistic regression models. **a** Model 1: multivariate logistic regression model of healthy OSAHS; (**b**) Model 3: multivariate logistic regression model of OSAHS with arterial hypertension. The area under the curve (AUC) was also shown

The sensitivity and specificity of these serum miRNAs as potential diagnostic and early warning markers in OSAHS patients were investigated by ROC analysis. The AUC of miR-126-3p in the two groups was 0.822 (95% confidence interval (CI) = 0.671–0.973; sensitivity: 86.7%; specificity: 66.7%; *P* = 0.0027), 0.871 (95% CI = 0.747–0.995; sensitivity: 86.7%; specificity: 73.3%; *P* < 0.001) (Fig. 6a). The AUC for let-7d-5p was 0.778 (95% CI = 0.598–0.958; sensitivity: 66.7%; specificity: 86.7%; *P* < 0.001) (Fig. 6b). The AUC for miR-320b was 0.751 (95% CI = 0.570–0.932; sensitivity: 86.7%; specificity: 66.7%; *P* = 0.019) (Fig. 6c). The AUC for miR-145-5p was 0.849 (95% CI = 0.711–0.986; sensitivity: 66.7%; specificity: 93.3%; *P* = 0.0011) (Fig. 6d). The AUC for miR-107 was 0.733 (95% CI = 0.552–0.915; sensitivity: 86.7%; specificity: 60%; *P* = 0.029) (Fig. 6e). The AUC for miR-26a-5p was 0.729 (95% CI = 0.543–0.914; sensitivity: 100%; specificity: 53.3%; *P* = 0.032) (Fig. 6f). Logistic regression analysis was used to evaluate the differentiating ability of the miRNAs in combination. A first multivariate model (model 1) (Table 3) was employed to identify the healthy OSAHS by using the expression levels of both miR-145-5p and let-7d-5p. Both of these miRNAs contributed significantly to the model as shown by the likelihood ratio test. Model 1 exhibited good differentiating ability (AUC = 0.951; 95% CI: 0.856 to 1.00; sensitivity: 93%; specificity: 100%; *P* < 0.001) (Fig. 7a). Model 2 only included miR-126-3p, given that it was not a miRNA specific to OSAHS with arterial hypertension, and among the subset of differentially expressed miRNAs, miR-26a-5p and miR-107 could increase the AUC of model, they were included in the final model (model 3) (Table 4). Figure 7b shows the ROC of the model 3 (AUC = 0.969; 95% CI: 0.915 to 1.00; sensitivity: 86.7%; specificity: 100%; *P* < 0.001). These models were shown to be able to well differentiate OSAHS patients from healthy controls.

**Table 3:** Multivariate logistic regression model of healthy OSAHS

Variables	Model 1	
	OR (95% CI)	*P* value
miR-145-5p	15.96 (1.69–151.2)	0.016
let-7d-5p	8.49 (1.47–48.93)	0.017

*OR* Odds ratio, *CI* Confidence interval

**Table 4:** Multivariate logistic regression models of OSAHS with arterial hypertension

Variables	Model 2		Model 3	
	OR (95% CI)	*P* value	OR (95% CI)	*P* value
miR-126-3p	118.81 (2.1–6738.68)	0.02	70.2 (1.04–2753.04)	0.04
miR-26a-5p	–	–	2.31 (0.4–13.33)	0.35
miR-107	–	–	2.76 (0.18–42.88)	0.46

*OR* Odds ratio, *CI* Confidence interval

## Discussion

As a serious poorly-understood clinical condition, OSAHS is associated with many diurnal symptoms and has been a subject of active studies [23–25]. So far, progress has been limited in spite of the continuous research efforts [26]. To find better diagnostic and therapeutic strategies, we need to fully understand its underlying mechanisms. Serum miRNA, due to its unique pattern of the expression, can serve a fingerprint for the evaluation of prognosis of various diseases. Previous studies prompted us to speculate that, by identifying dysregulated miRNAs, it is possible to find specific biomarkers of different subsets of OSAHS. In this study, we tried to find representative expression signatures in peripheral blood that might be associated with OSAHS, with an attempt to identify the early warning signs and prognostic markers of healthy OSAHS and OSAHS with arterial hypertension. Moreover, we theorized that the combination of several biomarkers may help achieve more accurate assessment of OSAHS.

In this study, we analyzed the differences in serum miRNA expression in healthy controls, healthy OSAHS and OSAHS with arterial hypertension using microarray technique, qRT-PCR and statistical analysis. Our data showed six serum miRNAs had significant changes in OSAHS patients as compared to healthy controls. A single cluster of miRNAs appears to be able to specifically identify healthy OSAHS and OSAHS with arterial hypertension. The specificity and sensitivity of the six miRNAs were not good in predicting OSAHS. We used a logistic regression analysis to select those miRNAs that were associated with the two subtypes of OSAHS. The results showed that the combination of miR-145-5p and let-7d-5p and the combination of miR-126-3p, miR-26a-5p and miR-107 was more valuable, in terms of specificity and sensitivity, in differentiating the OSAHS subtypes.

Among the dysregulated miRNAs, the miR-126-3p was found to be consistently down-regulated in healthy OSAHS and in OSAHS with arterial hypertension, and in both types of OSAHS, miR-126-3p had good predictive value, indicating that it, as a marker, was involved in the pathogenesis of both healthy OSAHS and OSAHS with arterial hypertension. MiR-126, one of the most abundant miRNAs in endothelial cells [27], has been found to regulate multiple processes, such as promotion of endothelial cell differentiation and maturation in embryonic vasculogenesis, inhibition of angiogenesis and proliferation of mature endothelial cells [28–30]. A study showed that miR-126 was down-regulated in cells under hypoxic conditions both in vitro and in vivo [31]. Meanwhile, mounting evidence indicates that endothelial function is impaired in OSAHS patients [32–34]. In addition, Kontaraki et al. found that expression of miR-126 was down-regulated in hypertensive patients compared to healthy controls [35]. Endothelial dysfunction and vascular remodeling are responses to the organ damage under hypertensive and hypoxic states. As an endothelium-specific factor, miR-126 could protect organs or tissues from hypoxia/reoxygenation (H/R)-induced injury [36] and inflammation, by enhancing endothelial repair during vascular remodeling [37]. Our results indicated that this miRNA might be involved in the specific regulation of endothelial function in OSAHS patients.

Interestingly, among specific miRNAs in the two groups, most of them were hypoxamiRs (hypoxia-associated miRNAs), a specific group of miRNAs that has been proved to be associated with hypoxia [38]. In OSAHS with arterial hypertension, miR-26a-5p, miR-107 were confirmed to be down-regulated. Several mechanisms have been suggested for the pathogenesis of hypertension, including increased activity of the sympathetic nervous system, overactivation of the renin-angiotensin-aldosterone system, dysfunction of the vascular endothelium, impaired platelet function, thrombogenesis, vascular smooth muscle and cardiac hypertrophy, and altered angiogenesis [14, 39]. These two miRNAs were reportedly dysregulated by hypoxia and were involved in cardiovascular disease, suggesting that they are involved in the pathogenesis of OSAHS with arterial hypertension. In fact, miR-26 has been consistently reported to be a contributor to endothelial dysfunction and impaired angiogenesis. One study found that miR-26a might exert an anti-apoptotic effect in endothelium by inhibiting TRPC6-induced calcium overload, and its expression was reduced in ApoE^−/−^ mice with atherosclerosis induced by high-fat diet [40]. Moreover, in another similar study, inhibiting miR-26a expression could promote apoptosis of vascular smooth muscle cells [41]. These studies demonstrated that, in endothelial cells, miR-26a possessed significant anti-apoptotic effect. Icli et al. showed that miR-26a acts as an anti-angiogenic factor, and the reduced miR-26a level could promote angiogenesis in endothelial cells [42]. Both miR-26a and miR-107 contributed to cell cycle arrest during oxygen deprivation that resulted in impaired cell proliferation [43]. Indeed, miR-107 blocked hypoxic signaling by suppressing HIF-1β expression, and mediated p53 regulation of hypoxic signaling and tumor angiogenesis [44]. In particular, miR-107 was a lipid-modulated miRNA that plays an important role in metabolic diseases. MiR-107 has been shown as a key regulator of insulin sensitivity [45], and was an important modulator in hepatic lipid metabolism [46]. A recent study exhibited that miR-107 could regulate circadian rhythm in cultured cells by targeting CLOCK genes. Moreover, the effect of miR-107 on lipid metabolism could be partially mediated by modifying the circadian system [47]. In addition, the miR-107-mediated regulation of intestinal microbiota and proinflammatory cytokines has been considered to be important for the maintenance of intestinal homeostasis [48]. Presumably, miR-107 might regulate a good many events involved in the development of OSAHS with arterial hypertension.

Meanwhile, let-7d-5p, miR-320b and miR-145-5p were validated to be the specific miRNAs in healthy OSAHS. It was reported that let-7 miRNA was one of the highly-expressed miRNA in the human brain [49]. Specifically, let-7 miRNAs was the first known human miRNA to play a vital role in brain development and could regulate neuronal differentiation/maturation, and nerve degeneration/regeneration [50–52]. More importantly, let-7 family members respond to hypoxia in a cell-specific manner [50]. Let-7d-5p was previously reported to be involved in the development of psychiatric diseases or degenerative disease of the central nervous system [53, 54]. Consistent with previous reports, our study showed that let-7d-5p miRNA was down-regulated in healthy OSAHS. Meanwhile, a large proportion of adults with OSAHS were found to suffer from one or more neurocognitive impairment(s), including excessive daytime sleepiness, fatigue, depressed mood, impaired memory, and/or poor concentration [55]. Accordingly, decreased serum let-7d-5p in OSAHS patients might account for the hypoxia-induced neural injury.

Among the miRNAs investigated in this study, cardiovascular-related miRNAs are of great value in the research of OSAHS. It has been reported that miR-320b was down-regulated during acute myocardial infarction and was intimately related to plaque stability [56, 57]. Previous studies suggested that miR-320b participates in a wide array of biological activities, including ischemia-reperfusion injury, angiogenesis [58, 59]. On the other hand, miR-320b exerts a significant effect on endothelial cells. During ischemia, the down-regulated miR-320b level could increase the release of endothelial vasoactive factors, such as VEGF, ET-1 and FN [60], thereby increasing the risk of atherosclerosis and ischemic cerebrovascular diseases. The coincidence between our finding and previous results suggests that miR-320b may cause cardiovascular and cerebrovascular disease in patients with OSAHS through recurrent hypoxia and reoxygenation. A previous study suggested miR-145 might be a regulator in vascular smooth muscle cells (VSMCs) both in vivo and in vitro, and might be a major miRNA that efficiently drove VSMC differentiation from multipotent stem cells [61]. A recent study exhibited that miR-145 was significantly suppressed and Smad3 was subsequently increased in hypoxia-treated VSMCs. Moreover, miR-145/Smad3 signaling pathway might promote OSAHS-induced aortic remodeling, which might be initiated by inflammation and oxidative stress [62]. In addition, hypoxia was found to aggravate inflammatory response and miR-145-5p might play an anti-inflammatory role to protect cells from ischemic and hypoxic injury [63]. It is well-known that, during sleep, intermittent hypoxia can cause sympathetic activation, evoke systemic inflammation, oxidative stresses and vascular dysfunction [64–66]. Meanwhile, we observed that the circulating miR-145-5p experienced significant changes, suggesting that healthy OSAHS are more vulnerable to cardiovascular diseases.

Microarray tends to yield a long string of target genes. GO and KEGG pathway analyses are generally employed to know their molecular functions and biological processes of these genes [21, 67, 68]. In this study, we used GO analysis and found that the miRNAs we singled out were correlated with nervous system differentiation/development, cancer development, metabolism, vascular regulation, and signal transduction. In addition, KEGG pathway analysis revealed that these miRNAs were involved in metabolism, circadian rhythm, receptor interaction, HIF-1 signaling pathway, neurotrophin signaling pathway, calcium signaling pathway. In previous studies conducted in OSAHS patients, anatomical and neuroimaging findings showed that the patients had nervous system damage. Moreover, hypoxia-inducible factor (HIF), as a key regulator of hypoxia response in cells and tissues, plays an indispensable role in hypoxia. Meanwhile, OSAHS was shown to be an independent risk factor for a variety of metabolic diseases. These may explain the link between the above-mentioned biological processes and OSAHS. Besides, according to the miRNA-gene-network, these genes targeted by the miRNAs were involved in hypoxia, nerve growth, metabolism, vascular endothelial differentiation. Especially, insulin-like growth factor-1/insulin-like growth factor 1 receptor (IGF1/IGF1R) were found to partake in nerve regeneration [52], regulation of the viability of schwann cells [69, 70] and the axon growth of motor neurons [71]. Another key gene was the angiomotin (AMOT), which plays an important part in angiogenesis [72]. More importantly, an OSAHS genome-wide gene expression array study showed that AMOT was involved in OSAHS-related excessive daytime sleepiness by regulating a variety of endothelial cell functions [73].

However, several limitations of the present study should be mentioned. First, the sample size of our study was relatively small. We sub-grouped OSAHS patients in terms of freedom of complications and presence of arterial hypertension only, which could substantially limit number of available subjects. Many adult OSAHS patients often had more than one complication, such as diabetes, hypertension, dyslipidemia or cardio-cerebrovascular diseases. On the other hand, absence of complications and the single complication in our series increased the power of our study. Numerous therapeutic studies have shown that early OSAHS damage were reversible [74]. Study of these special types of OSAHS help us better understand the progression of the diseases. Another limitation was that in comparing controls and OSAHS with arterial hypertension, let-7d-5p was down-regulated in the training set, but was significantly up-regulated in the validation set. The results of qRT-PCR and microarray were obviously inconsistent. We believe this inconsistency had something to do the small sample size of microarray, indicating that the results of microarray still needed to be further validated in a larger samples. In addition, during the subsequent verification, the miRNAs we selected were interpreted primarily on the basis of microarray results and previous findings, a number of miRNAs with low expression levels might have been left out. Regardless of these limitations, our study provides direct evidence that dysregulated miRNAs are important contributors to OSAHS progression. Moreover, the target genes of these six miRNAs and the potential molecular mechanisms may provide useful information for the treatment of OSAHS.

## Conclusion

Our study demonstrated that, the miRNA expression was dysregulated in OSAHS patients, and some miRNAs were differentially expressed in healthy OSAHS and OSAHS with arterial hypertension. These miRNAs might be used as biomarkers for stratifying OSAHS patients, achieving early diagnosis and improving diagnostic accuracy.

## Abbreviations

AASM: American Academy of Sleep Medicine
AHI: Apnea hypopnea index
AMOT: Angiomotin
ANOVA: One-way analysis of variance
AUC: Area under the curve
BMI: Body mass index
CI: Confidence interval
FDR: False discovery rate
HIF: Hypoxia-inducible factor
ICSD-II: International Classification of Sleep Disorders
IGF1/IGF1R: Insulin-like growth factor-1/insulin-like growth factor 1 receptor
OR: Odds ratio
OSAHS: Obstructive sleep apnea-hypopnea syndrome
PSG: Polysomnography
qRT-PCR: Quantitative real-time PCR
ROC: Receiver operating characteristic
VSMC: Vascular smooth muscle cell
